# Automated transition analysis of activated gene regulation during diauxic nutrient shift in *Escherichia coli* and adipocyte differentiation in mouse cells

**DOI:** 10.1186/s12859-018-2072-y

**Published:** 2018-05-08

**Authors:** Yoichi Takenaka, Kazuma Mikami, Shigeto Seno, Hideo Matsuda

**Affiliations:** 1 Faculty of Informatics, Kansai University, Ryousenji 2-1-1, Takatsuki, Osaka, Japan; 2 Graduate School of Information Science and Technology, Osaka University, Yamadaoka 1-5, Suita, Osaka, Japan; 3 Graduate School of Medicine, Osaka University, Yamadaoka 2, Suita, Osaka, Japan; 4 Recruit Holdings Co. Ltd., Marunouchi 1-9-2, Chiyoda, Tokyo, Japan

**Keywords:** Gene regulatory network, Network dynamics, Time course, Cell differentiation, Adipocyte

## Abstract

**Background:**

Comprehensively understanding the dynamics of biological systems is among the biggest current challenges in biology and medicine. To acquire this understanding, researchers have measured the time-series expression profiles of cell lines of various organisms. Biological technologies have also drastically improved, providing a huge amount of information with support from bioinformatics and systems biology. However, the transitions between the activation and inactivation of gene regulations, at the temporal resolution of single time points, are difficult to extract from time-course gene expression profiles.

**Results:**

Our proposed method reports the activation period of each gene regulation from gene expression profiles and a gene regulatory network. The correctness and effectiveness of the method were validated by analyzing the diauxic shift from glucose to lactose in *Escherichia coli*. The method completely detected the three periods of the shift; 1) consumption of glucose as nutrient source, 2) the period of seeking another nutrient source and 3) consumption of lactose as nutrient source. We then applied the method to mouse adipocyte differentiation data. Cell differentiation into adipocytes is known to involve two waves of the gene regulation cascade, and sub-waves are predicted. From the gene expression profiles of the cell differentiation process from ES to adipose cells (62 time points), our method acquired four periods; three periods covering the two known waves of the cascade, and a final period of gene regulations when the differentiation to adipocytes was completed.

**Conclusions:**

Our proposed method identifies the transitions of gene regulations from time-series gene expression profiles. Dynamic analyses are essential for deep understanding of biological systems and for identifying the causes of the onset of diseases such as diabetes and osteoporosis. The proposed method can greatly contribute to the progress of biology and medicine.

## Background

Acquiring a comprehensive understanding of biological systems dynamics is among the biggest challenges in biology and medicine. The processes of all organisms (including humans) are time-variant. Cells cycle and divide, change their internal states and differentiate into other cell types when stimulated from the outside. Abnormal cell differentiation causes diseases and cancer. Dynamic systems are universal in organisms.

Biological systems dynamics can now be understood through advanced biological technologies. On-chip hybridization and DNA sequencing has enabled the simultaneous measurement of the expression levels of many genes. Many of the accumulated data are registered in public databases such as GEO (Gene Expression Omnibus) [1, 2], Array Express [3] and Sequence Read Archive [4]. Special-purpose databases are also accessible. The references of mammalian tissue gene expressions are stored in RefEx (Reference Expression Dataset) [5], whereas the data from human tissues, cell lines and cancers are distributed through the Human Protein Atlas [6]. Mouse data are archived in the specialized database GXD (Gene expression database) of the Mouse Genome Informatics resource [7]. These databases are freely accessible to all users.

The above databases store the time-course movements of the gene expression levels in multiple organisms. For example, a search for “*time-course*” (2017/7/12) returned 913 datasets in GEO and 7623 entries in SRA. Based on these data, researchers have revealed the biological mechanisms of processes such as cell cycling [8] and diauxic shifts [9]. The adipogeneses of mice and humans have also been compared using time-course profiles [10]. These studies cover a broad range, from biology, through medicine, to pharmacology.

**Fig. 1:**
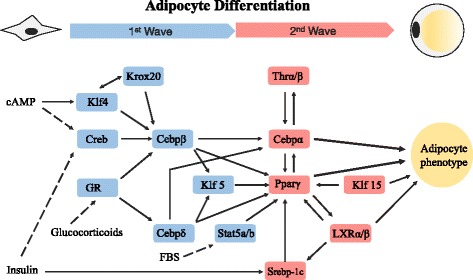
The gene regulatory network of murine cell differentiation into adipocytes occurs in two waves (distinguished by different colors) [26]

Numerous systems operate in living cells. Examples are metabolism, genetic and environmental information processing, cellular processes, and organismal systems [11]. Concurrent functioning of these systems in vivo maintains the life activities of the cell. Systems have been extensively researched and summarized as pathways in the KEGG (Kyoto Encyclopedia of Genes and Genomes) database [11]. Control of gene expression levels is another life-sustaining system. Gene regulation defines the relation between a controlling agent and its target gene. The gene regulatory system can be represented as a network of nodes (genes) connected by directed edges (regulation of a downstream gene by an upstream gene). The gene regulatory networks of model organisms are available in databases such as RegulonDB [12, 13], which contains the nearly-complete gene regulation of *Escherichia coli*, and the yeast network databases YEASTRACT [14–17]. The FANTOM projects have attempted to exhaustively reveal the relations between mouse and human in many tissues [18–22]. Gene regulatory networks are also provided in commercial databases such as TransFac [23, 24] and Quiagen’s Ingenuity Pathway Analysis [25]. The regulatory relations between genes, at least in the model organisms, will be revealed exhaustively in the near future. However, although databases provide numerous gene regulations, the relationships are basically static. Each database entry supplies a gene regulatory relation and the experimental environment in which the relation was activated. Very few entries include the chronological order of the activations, despite our increasing knowledge of signaling pathway cascades. When the chronological order is given, the experimental environment includes the cell cycles and diauxic shift of the nutrient source. Adipocyte differentiation is accompanied by a rough sketch of the transition of active expression controls [26] (see Fig. 1). The differentiation involves a single transition point and two waves of activated gene regulations, although more than two waves have been suggested [26]. The dynamics of gene-expression control is a new research topic, with many unknowns at present.

Gene regulation activity can be dynamically analyzed from gene expression profiles. Given time-course data, researchers have sought the transformations in the gene regulatory network through models, such as ordinary differential equations and graphical models. Ordinary differential equations can explain the activity level of a gene regulation. The explanatory and objective variables are the expression levels of the upstream genes and the downstream genes, respectively [27–29]. Graphical models describe the control relations in the gene expression as a network of nodes (genes) with directed edges (controls). The dynamics of biological systems can be analyzed by a graphical Gaussian model with a Markov property [30, 31], which measures the independence of any two genes using partial correlations.

Other graphical models are the gene relevance network and the Bayesian network. The former generates a gene regulatory network from the mutual information, and measures the degree of association between gene expression levels [32–34]. This model can also manage time-course gene expression profiles [35].

In the Bayesian network, a directed edge represents the probabilistic relationship between two genes by information-theoretic approaches. The plausibility of the network is measured by the Bayesian information criterion and the minimum description length [36, 37]. Bayesian networks have been extended to time-course data by introducing Markov properties into the joint probability distribution. The time-dependent Bayesian network is called a dynamic Bayesian network [38, 39].

**Fig. 2:**
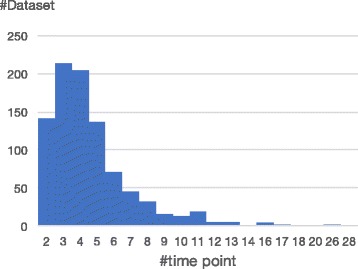
Number of time points in datasets returned by a “time course or time-course” query in the Gene Expression Omnibus. The average, mean and standard deviation of the distribution are 4.66, 4 and 2.82, respectively

The above-described methods have analyzed not only the gene expression profiles, but also general data. Researchers have obtained meaningful results from all data except time-course gene expression profiles, which are most severely limited by the small number of time points. Figure 2 shows the distribution of time points in the GEO database. There are 913 datasets with time-series expression profiles. The average, mean and standard deviation of the time points are 4.66, 4, and 2.82, respectively. The number of time points is far below the numbers of other common data. To clarify this fact, Table 1 lists the number of data points in benchmark problems for inferring causal relationship networks [37, 40–44]. All of these benchmarks are included in bnlearn of the R package [44]. Comparing Fig. 2 and Table 1, we observe that the number of time points is two orders of magnitude lower in the time-series expression profile than in the benchmarks. This data limitation not only prevents the estimation of correct relationships, but also stifles the dynamic analyses.

**Table 1:** Numbers of samples and variables in benchmark problems for inferring causal relationship networks

Benchmark	#Samples	#Variables
Learning.test	5000	6
Cigaussian.test	5000	8
Asia	5000	8
Alarm	20000	37
Insurance	20000	27
Hailfinder	20000	56

**Fig. 3:**
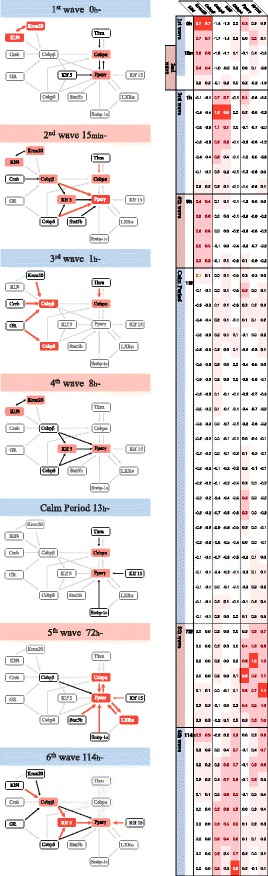
Six waves and one calm period during adipocyte cell differentiation. The tables show the strengths of the gene regulations calculated by [45]. Eight genes are regulated by other genes, and the strength changes between 0 and 196 h (white and red cells denote low and high strengths of the gene regulation, respectively). The period of each wave is also shown in the table. The left side of the figure presents the active gene regulations in each wave. The waves before and after the calm period correspond to the two waves reported in [26]. This analysis was performed by Takenaka and presented at Recomb 2016 [51]

Takenaka et al. [45] proposed a method that captures the dynamic changes in gene expression control from time-course expression profiles with a small number of time points. Their method quantifies the intensity at which genes control the expression levels of other genes. The temporal resolution of intensity is one time point (the observation unit of the time-course gene expression profile). Single time-point resolution is finer than the resolutions of other methods [46–49]. Assuming a profile with N time points, the method generates N sub-profiles, each omitting one time point. The effectiveness of the method was confirmed in the diauxic shift of *E.coli* [9] and the adipocyte cell differentiation in mouse tissue [45, 50, 51]. The results detected the time of the diauxic shift in *E. coli*, and two waves of active regulations during adipocyte cell differentiation (Fig. 3). The authors concluded that the method effectively reveals the dynamics of gene regulations. But the method does not have any procedure how to determine the number of breakpoints and when the breaks happen. Therefore, finding the breakpoint of active gene expression control by this approach is somewhat subjective, and is undeniably influenced by already known biological findings. In this research, we propose a mechanical method that eliminates this subjectivity.

## Method

We propose a method that analyzes the dynamics of gene regulations from time-series gene expression profiles. Here, the dynamics of gene regulations take two meanings. The first interpretation considers the dynamics of each gene regulation; whether the control of gene expression level by other genes is activated or inactivated at each time point in the time-series data. The second considers the dynamics of the gene regulatory network; the periods and pairs of their start and end times. The periods (sometimes called states, waves, or phases) are often biologically meaningful. The most well-known period is the cell cycle, which has two states; interphase and cell division. The interphase is divided into three sub-states called phases (Gap1, Synthesis, and Gap2), each with sub-phases. The cell cycle has four phases in total. Under these dynamics, the number of periods in a cell cycle is not fixed. The same situation occurs in other biological processes. Therefore, determining the number of periods in a biological process is a difficult task, and the dynamics of the gene regulatory network are difficult to analyze, especially in a manual analysis.

### Notations

To analyze the dynamics of gene regulations in the two interpretations by an automated approach, we formulate the analysis as an optimization problem. The notations of the formulation are listed below.

- ***Exp***: time-course gene expression profiles with |*T*| time points and |*G*| genes.
- ***T***: a set of time points in *Exp*. If *i*<*j*, then *t*_*i*_∈*T* is earlier than *t*_*j*_∈*T*.
- ***G***: a set of genes in *Exp*.
- ***M***: a two-dimensional matrix |*G*|×|*T*|, where *M*_*ij*_ represents the intensity at which the expression of gene *g*_*i*_∈*G* is controlled by others at time *t*_*j*_∈*T*. *M* is calculated from *Exp* by the method proposed in [45].

In our method, the activity of gene regulation is represented by a binary number, with 1 and 0 denoting active and inactive, respectively. The notations of the gene-regulation dynamics are explained below.

- *a*_*g*_: a binary vector of length |*T*| representing the activity of gene *g*∈*G* at time *t*_*j*_∈*T*.
- ***Act***: a binary matrix of size |*G*|×|*T*|, where *Act*_*i*_ equals $a_{g_{i}}$.
- ***Tra***: a subset of |*T*| representing the breaks between periods. For example, if *Tra*={*t*_3_}, the system has two periods; the first ending at *t*_3_ and the second starting at *t*_4_. If *Tra* is an empty set, no transition of the gene regulation occurs throughout the period.

### Score function

As previously mentioned, we analyze the dynamics of gene regulations by solving the optimization problem; that is, by finding the most plausible dynamic model of the gene regulations. This model is conditional on the activity of each gene and the model complexity. Regarding the first condition, a positive *M*_*ij*_ indicates high likelihood of activating the gene *g*_*i*_ at time point *t*_*j*_. The second conditional expression describes the penalty term of the Bayesian information criterion. Here, the complexity of the model is defined as the number of periods in the model, which deals with the trade-off between the goodness of fit to the model and the tractability of the model.

To find a plausible model for *M*, we must optimize the following score:

1 $$ \text{Score}(Act) = \sum_{i=1}^{|G|} \sum_{j=1}^{|T|} M_{ij} \cdot {Act}_{ij} - |Tra| \cdot \ln (|G| \cdot |T|),  $$

where *Tra* is calculated from *Act*. A time point *t*_*i*_∈*T* is a member of *Tra* if ∃*jAct*_*ij*_≠*Act*_*i*(*j*+1)_.

### Algorithm

The algorithm that finds the most plausible dynamic model of gene regulation is described below.

- **Input** a Matrix *M* calculated from the gene expression profile and a gene regulatory network by the method in [45].
- **Output** a Matrix *Act*, a set *Tra*, and a *score*.
- **Measure** Maximize the score function Score (*Act*).
- **Variables**
  *k*: The integer *k* represents the number of breaks between the periods in the model.
  *V*: A set of binary vectors of length |*T*|. Each element of *V* represents the activity of a gene regulation.

- **step 1** Initialize *k*←0 and *Tra*←{}
- **step 2** Generate a set of vectors *V* by the following sub-steps.
  1. add a zero vector to *V*
  2. add a one vector to *V*
- **step 3** *score*_0_←0
- **step 4** for each *g*_*i*_∈*G*, select a vector *v*∈*V* that maximizes the *g*_*i*_ score in the following sub-steps.
  1. select a vector *v* in *V* that maximizes *M*_*i*_·*v*^*t*^, where *M*_*i*_ is the *i*-th row of *M*.
  2. *score*_0_ +=*M*_*i*_·*v*^*t*^
  3. insert vector *v* into the *i*-th row of *Act*; *Act*_*i*_←*v*.
- **step 5** *score*_*best*_←*score*_0_; *Act*_*best*_←*Act*; *Tra*_*best*_←*Tra*
- **step 6** *k*+=1; If *k*=|*T*|, goto **Step 9**.
- **step 7** for each *Tra*={*t*|*t*∈*T*}, where |*Tra*|=*k*,
  - **step 7-1** generate a set of vectors *V* as follows:
  1. for each period *i* of *Tra*, generate a set *V*_*i*_ with two elements; a zero vector and a unit vector with lengths equal to that of the period.
  2. generate *V* as the direct product of all *V*_*i*_.
  - **step 7-2** *score*_*k*_←−2·*k*· ln(|*T*|·|*G*|)
  - **step 7-3** for each *g*_*i*_∈*G*, select a vector *v*∈*V* that maximizes the score by the following sub-steps.
  1. select a vector *v* in *V* that maximizes *M*_*i*_·*v*^*t*^, where *M*_*i*_ is the *i*-th row of *M*.
  2. *score*_*k*_ +=*M*_*i*_·*v*^*t*^
  3. insert vector *v* into the *i*-th column of *Act*; *Act*_*i*_←*v*.
  - **step 7-4** if *score*_*k*_>*score*_*best*_, *score*_*best*_←*score*_*k*_; *Act*_*best*_←*Act*; *Tra*_*best*_←*Tra*.
- **step 8** if *score*_*k*_>*score*_*k*−1_, goto **Step 6**.
- **step 9** output *Act*_*best*_, *Tra*_*best*_ and *score*_*best*_.

The above algorithm is a brute-force search with poor computational efficiency. However, as the gene expression profile contains very few time points, the above algorithm satisfies our requirements.

For larger datasets, the calculation time can be shortened by using a hash function from a gene and a period to the sub-score. Meanwhile, the computational complexity can be reduced by dynamic programming.

## Results and discussion

The effectiveness of the proposed method was tested on two biological datasets. The results of our method were also compared with a manual analysis of the periods in the time series. The first dataset contains the time-course gene expression profiles during the diauxic shift from glucose to lactose metabolism in *E. coli*. In this test, the method was assessed by its ability to detect the time of the diauxic shift.

The second dataset reports the adipocyte cell differentiation in the house mouse *Mus musculus*. Two periods (called waves in [26]) of gene regulation have been identified during this differentiation, and more waves are expected. This test evaluated whether the method can detect three or more plausible periods.

### Diauxic shift

#### Material

The first dataset is the time-course gene expression profile GSE7265 in the GEO dataset. The diauxic shift was observed in *E. coli* MG1655 and isogenetic mutants cultured in a medium containing glucose and lactose [9]. The expression levels of the genes were measured in oligonucleotide microarrays containing 70-base oligonucleotide probes. The wild-type profile contains 17 time points from 780 min. to 1089 min. after inoculation. At each time point, the triplicated expression values were averaged to obtain the representative expression level.

The time points are divided into two phases; the first lasting from 780 min. to 939 min., the second from 969 *min*.*to* 1089 min. The diauxic shift from glucose to lactose has been recognized in the first half, but information on the second half is completely lacking. Although the GS2765 gene expression profile contains 17 time points, the original paper [9] trimmed the profile to 10 time points (830-939 min. post-inoculation), and reported the growth ratio in the first half only. Therefore, the full dynamics of the diauxic shift can only be surmised.

The gene regulatory network of the diauxic shift comprises 31 genes and 50 gene regulations [45]. Fourteen enzymes are related to the glycolytic and lactose metabolic pathways. According to RegulonDB [12, 13], the expression levels of the enzymes are controlled by 14 transcription factors (TFs), and 50 TF–enzyme interactions are known. The present study adopts the gene list and the gene regulatory network used in [45].

The proposed method was implemented in R, and the matrix *M* representing the intensities of the gene regulations was calculated by an R package from GitHub https://github.com/takenakayoichi/tacs. The computed matrix *M* was input to the proposed method.

#### Results

**Fig. 4:**
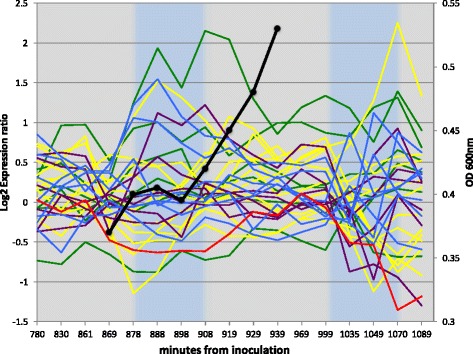
Expression levels of 31 genes (colored plots) and growth ratio (solid black line) during diauxic shift in *E. coli*. Vertical lines delineate the periods of the dynamic model detected by the proposed method

The proposed method detected five periods during the diauxic shift of wild type *E. coli*. The maximum score during each period is tabulated in Table 2. Although the proposed algorithm identified 6 periods, the maximum score was recorded over 8 periods for reference. The score peaks during Period 5 and the between-period breakpoints are 878 min., 908 min., 999 min. and 1049 min. The first, second and third periods belong to the first half of the diauxic shift; the remainder belong to the second half. The periods are also indicated by the vertical lines in Fig. 4.

**Table 2:** Maximum scores in each period of diauxic shift in *E. coli* (central column) and differentiation to murine adipocytes (right column) (maximum scores are in bold)

#Period	Diauxic shift	Adipocyte
1	16.42	1.50E-13
2	23.57	50.38
3	26.23	70.30
4	28.00	**74.76**
5	**28.44**	73.41
6	26.81	72.02
7	25.02	69.25
8	23.14	67.87

Takenaka et al. [45] manually detected three periods in the GS2765 gene expression profile, with breakpoints at approximately 888 − 908 min. and 1070 − 1089 min. The breakpoints were expressed as ranges because they were difficult to pinpoint by the manual approach. In their analysis, the first and second periods belonged to the first half of the diauxic shift, and the third period belonged to the second half.

#### First half

Our method divided the first half of the experiment into three periods. Figure 4 shows the growth ratios in each period. During the first and third periods, the *E. coli* exist in the logarithmic growth phase and are consuming glucose and lactose, respectively. The second period is consistent with cessation of cell proliferation.

In comparison, the manual curation identified only two periods in the first half. The division point was exactly centered in the growth arrest period. From the difference between our results and the manually curated results, we can identify whether a mechanism that regulates the gene expression level exists independently in the logarithmic and growth-arrest phases.

Which of these methods expresses more realistic biological properties? Manual curation suggests that lactose consumption begins immediately after glucose depletion. On the other hand, the proposed method suggests that once the glucose is depleted, the cells cease growing while seeking a new nutrient source, and resume growth when an alternative nutrient is found. We consider that the proposed method presents a more plausible dynamic model than manual curation. One expects that the *E. coli* cells cannot immediately access a new nutrition source once the original source is depleted. If this were possible, logarithmic growth would continue without interruption.

#### Second half

The second half of the experiment was continuous in the manual curation, but divided into two periods in the proposed method. Once the *E. coli* had consumed the glucose and lactose in their nutrient medium, they began seeking the next nutrition source. When no further source was found, the gene expression regulations changed sufficiently for detection by both methods. However, as the expression profile provides the only available information in the second half, the actual number of periods in the second half of the experiment is difficult to judge.

### Adipocyte differentiation

#### Materials

The second dataset was a time-course gene expression profile of mouse adipocyte differentiation. The RNAs in this dataset were collected from mouse ST2 bone marrow stroma cell-derived stem cells (RCB0224) obtained from the RIKEN BioResource Center (BRC, Tsukuba, Japan). ST2 cells were induced by changing the medium from RMPI1640 to DMEM supplemented with 10% FBS, 0.5 mM 3-isobutyl-1-methylxanthine (MIX), 0.25 *μ*M DEX, and with insulin-transferrin-selenium-X supplement containing 5 *μ*g/ml of insulin and 1 *μ*M rosiglitazone. After 48 hours, the differentiation medium was replaced with DMEM supplemented with 10% FBS. The RNAs collected at 62 time points during adipocyte differentiation were measured using an Affymetrix GeneChip Mouse Genome 430 2.0 Array. The time points were 0, 5, 15, 30 and 45 min., hourly from 1 to 30 h, and 6-hourly from 36 to 192 h after adipogenesis induction. Each datum was background-subtracted and normalized in the robust multi-array analysis (RMA). The gene expression profiles are available from the Genome Network Platform [50].

Previous research has unveiled the important regulators of adipocyte development and a two-wave cascade in this process [26] (see Fig. 1). The network includes 24 regulations between 14 genes, but undetected sub-waves have been predicted [26].

**Fig. 5:**
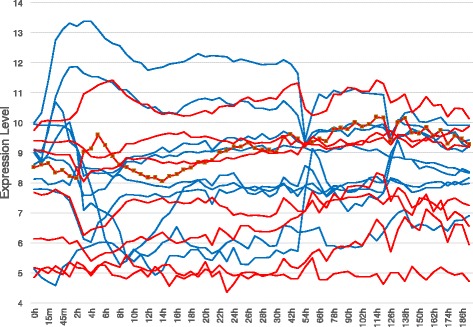
Expression Levels of 14 genes during murine adipocyte differentiation. Blue and red lines indicate the expression levels of genes belonging to the first and second waves in Fig. 1, respectively. The red line with square markers plots the expression levels of pparg, a marker gene of adipose cells

The expression levels of the 14 genes are shown in Fig. 5. Among them, the line with square indicates pparg, a known marker of adipocyte cells. The expression level of pparg increases from 2 to 6 h and then decreases. The decline is followed by a gradual rise after 14 h.

**Fig. 6:**
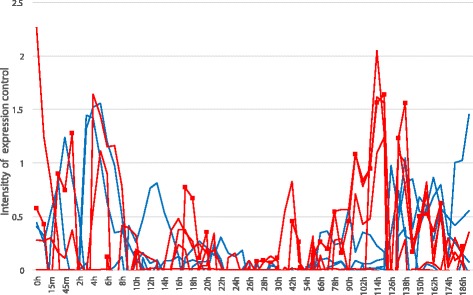
Intensity of genes whose expression levels are controlled by other genes. Blue and red lines show the intensities of genes belonging to the first and second waves in Fig. 1, respectively. The red line with square markers plots the intensity of pparg, a marker gene of adipose cells

Figure 6 shows the intensities of the expression levels controlled by other genes, which constitute the intensity matrix *M* of our algorithm. The intensities were calculated from the gene expression profiles in Fig. 5 and the gene regulatory network in Fig. 1.

#### Result

**Fig. 7:**
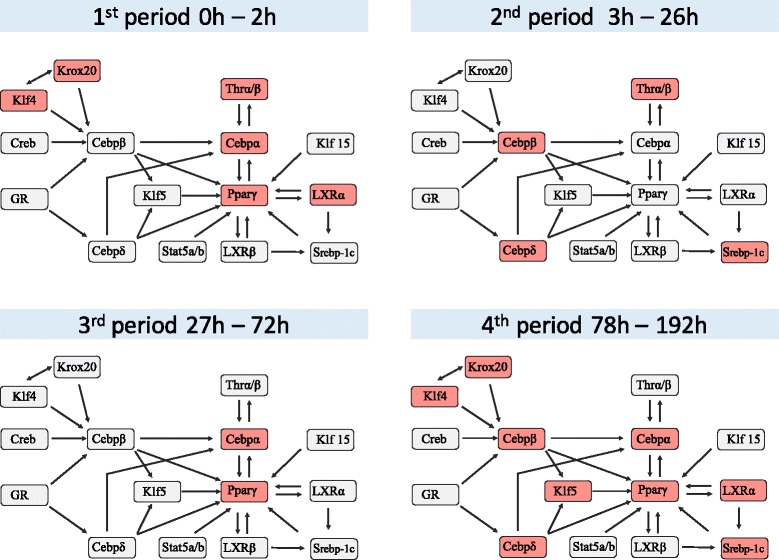
The four periods of differentiation into murine adipocytes detected by the proposed method. Dark- and light-shaded boxes enclose genes whose expression level is controlled and not controlled by upstream genes, respectively

The proposed method detected four periods of adipocyte differentiation. The maximum score in each period is listed in Table 2. The algorithm terminated after five periods, but 8 periods are included for referencing purposes. The score peaks during period 4, and the between-period breakpoints are 2 h, 26 h and 72 h. Figure 7 shows the dynamics of the gene regulatory network. In the figure, we classify the gene whose expression level during a period is controlled by upstream genes or not by the average of the intensity during the period is larger than zero or not.

The two waves of the known cascade in Fig. 1 appear in the four periods detected by our algorithm. During the first period of adipocyte differentiation, the expression controls of the most upstream genes (Klf4 and Knox20) are activated. During the second period, the expressions of Cebp *β* and Cebp *δ* are controlled and the regulations of the second wave (Thr *α*/*β* and Srebp-1c) begin activating. The pparg and cebp *α* genes are regulated during the third period, and the most downstream genes are regulated during the fourth period. The four-period differentiation identified by our method includes the known waves, but appears to be more precise.

The four periods detected by our method differ from the two-wave differentiation in two ways. First, the regulations of Pparg and Cebp *α* are activated twice. Once at the first period, and re-activated in the second wave composed of third and fourth periods. Pparg is a marker of adipose cells and a known regulator of adipocyte differentiation. The two activations of the pparg gene are confirmed in Fig. 5. The expression level of Pparg peaks at 6 h and 114 h. Most of the gene expression regulations are activated in the fourth period. By the start time of this period (78 h), the cells will have produced lipid droplets [52]. Therefore, we consider that the differentiation into adipocytes was completed during the fourth period, and was stabilized thereafter. It appears that many regulations of the adipocyte-related gene expression levels are activated in the fourth period.

## Conclusions

We proposed a method that detects the dynamics of gene regulatory networks at the temporal resolution of single time points in the underlying gene expression profiles. The dynamics are modeled as periods of activated regulations. The plausibility of the model was quantified using the Bayesian information criterion. The problem constitutes a combinatorial optimization problem that find the highest-scoring model. The algorithm inputs the gene expression profiles and a gene regulatory network, and returns the activated regulations, divided into periods.

The effectiveness of the method was validated in two datasets; the diauxic shift from glucose to lactose in *E. coli* and adipocyte differentiation in the mouse. In both datasets, the proposed method detected more plausible dynamic models than existing models. We believe that the proposed method can precisely reveal the dynamics of biological systems.
